# Biomonitoring of Environmental Phenols, Phthalate Metabolites, Triclosan, and Per- and Polyfluoroalkyl Substances in Humans with Chromatography and Mass Spectrometry

**DOI:** 10.3390/toxics13121029

**Published:** 2025-11-28

**Authors:** Anastasia Chrysovalantou Chatziioannou, Antonis Myridakis, Euripides G. Stephanou

**Affiliations:** 1 Nutrition and Metabolism Branch, International Agency for Research on Cancer, 69366 Lyon CEDEX 07, France; 2 Division of Systems Medicine, Department of Metabolism, Digestion and Reproduction, Imperial College London, London W12 0NN, UK; a.myridakis@imperial.ac.uk; 3 Department of Chemistry, University of Crete, Voutes University Campus, 70013 Heraklion, Greece; evris.stephanou@uoc.gr

**Keywords:** biomonitoring, endocrine disruptors, human biofluids, chromatography, mass spectrometry, chemical analysis

## Abstract

Endocrine-disrupting chemicals (EDCs) are widespread organic compounds that interfere with hormone signaling and are linked to reproductive, developmental, cardiovascular, and cancer-related health effects. Key EDCs include bisphenol-A and its analogs, phthalates, parabens, triclosan, and per-and polyfluoroalkyl substances (PFAS), which are commonly present in personal care products and plastics. Human exposure occurs via environmental exposure through ingestion, inhalation, and dermal contact, with persistent compounds such as PFAS accumulating in blood, while others are excreted in urine as free or conjugated metabolites. Accurate assessment of EDC exposure, particularly during pregnancy and early childhood, requires robust analytical methods. Liquid and gas chromatography coupled with mass spectrometry (LC-MS and GC-MS) are the most widely used techniques to date. LC-MS is favored for its sensitivity, specificity, and minimal sample preparation, whereas GC-MS provides adequate performance but often requires time-consuming derivatization. This review summarizes current LC-MS and GC-MS methodologies for multi-class EDC biomonitoring, emphasizing sample preparation, analyte coverage, and methodological strengths and limitations, providing a practical reference for human exposure studies using common biological matrices such as urine and blood.

## 1. Endocrine-Disrupting Chemicals

Endocrine-Disrupting Chemicals (EDCs) are a group of organic compounds that cause significant alterations to normal hormone function in humans and wildlife. EDCs interfere with hormone biosynthesis, metabolism, or action, leading to a deviation from normal homeostatic control or reproduction in humans [1]. They disrupt the endocrine system by competing with naturally occurring hormones, such as estradiol, or by altering the synthesis and metabolism of these hormones [2]. Furthermore, there is strong evidence of reproductive toxicity in laboratory animals and potential health effects in humans, including reproductive and developmental problems, cardiovascular diseases, and cancer [3,4,5]. The hydrophilic species are often extensively metabolized and further react mainly with glucuronide and sulfate groups through the detoxification mechanisms of human body, finally forming stable conjugates in varied proportions which are excreted via urine [6,7,8]. In order to assess the exposure to the initial chemicals, the stable conjugates are hydrolyzed enzymatically and the total levels, which are in the region of low ng/mL, are estimated by monitoring only the free species in the hydrolyzed urine [9,10]. The advantages of this approach include, apart from the experimental simplicity of a smaller target analyte list, the tackling of the scarcity/total absence of commercially available conjugate standards. The lipophilic species on the other hand do not undergo extensive chemical changes upon entering human body and in most cases bioaccumulate; exposure assessment can be performed by measuring them in blood. PFAS that circulate almost exclusively as the parent anionic acids also belong in this category. PFAS show minimal biotransformation and are mostly bound to serum proteins (mainly albumin). Therefore, PFAS biomonitoring targets the total unconjugated form in serum/plasma mainly, since long-chain PFAS are poorly excreted in urine, and they have long elimination half-lives [11,12].

**Figure 1:**
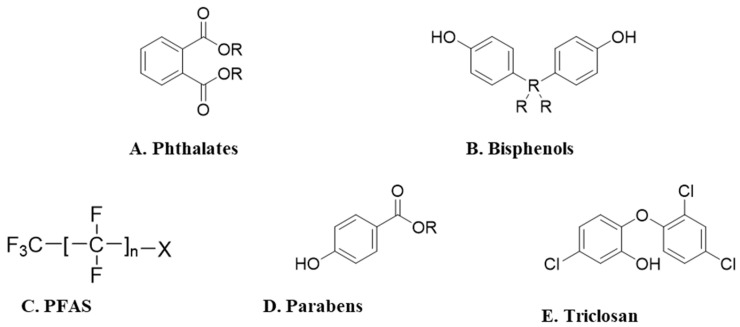
General chemical structures of the studied endocrine disruptors.

Bisphenol-A (BPA) and other bisphenol substitutes (such as BPF, BPP, BPS, and BPAF), parabens (PBs), phthalates (PEs), triclosan (TCS), and per- and polyfluoroalkyl substances (PFAS) are well-recognized endocrine-disrupting chemicals (EDCs) (Figure 1).

Approximately ten million tons of BPA are produced annually worldwide [13], and safety concerns over BPA have led to the widespread use of bisphenol substitutes, which have controversial toxicological profiles [14,15]. Phthalates, with a global market share of ten billion US dollars in 2020, are one of the world’s highest-production chemical families [16]. Parabens, excluding water, are considered the most abundant cosmetic ingredient and are found in approximately 80% of personal care products [3]. PFAS are a large class of highly persistent chemicals. Due to their oil-, water-, and stain-resistant properties, PFAS have been commonly utilized since the 1950s in diverse categories of consumer products, such as food packaging, cosmetics, and clothing [17].

Human exposure to these chemicals occurs through the environment, food intake, and the use of products containing them or coming into contact with them, via inhalation, dermal contact, and ingestion [1,3,5,13,18].

### 1.1. Bisphenols

2,2-Bis(4-hydroxyphenyl)propane, more commonly known as bisphenol-A (BPA), is the most extensively studied compound in this category. It is widely used in the industry for the production of various pesticides, epoxy resins, and polycarbonate plastics. BPA can be found in food and beverage processing, as well as in various products such as dental sealants, personal care products, baby drinking bottles, building materials, flame retardant materials, optical lenses, materials for the protection of window glazing, canned food, and household electronics. Especially when heated, BPA readily migrates from polycarbonate plastic or epoxy resins, for example, during microwaving food in plastic packaging [13]. BPA has shown anti-estrogenic and anti-androgenic effects on the hypothalamic–pituitary–gonadal hormone feedback system [19]. Human exposure to BPA has been associated with several adverse health effects, including cardiovascular diseases, brain and behavioral effects, obesity, diabetes, liver abnormalities, reproductive issues, and alterations in thyroid function [13]. BPA is excreted within several hours of exposure, mainly via urine, in its more hydrophilic glucuronide or sulfate conjugate forms, and partially in its free form [20].

Other bisphenol analogs with similar structures, such as 4,4′-dihydroxydiphenylmethane (BPF), 4,4′-sulfonyldiphenol (BPS), 4,4′-(1,4-phenylenediisopropylidene)bisphenol (BPP), and 4,4′-(hexafluoroisopropylidene)diphenol (BPAF), have started being widely used in recent years due to concerns about the health effects associated with BPA [14]. However, they have also attracted the attention of the scientific community due to their endocrine-disrupting chemical (EDC) activity and potentially adverse health outcomes [21,22]. Similarly to BPA, they are partially metabolized to glucuronates or sulfated analogs and mainly excreted via urine, where their levels serve as exposure biomarkers [10].

### 1.2. Parabens

Parabens (PBs) are a group of alkyl esters of p-hydroxybenzoic acid, characterized by their low production cost, high chemical stability, inertness, and low acute toxicity. These properties make them widely used in industry as antimicrobial preservatives against mold and yeast in cosmetics, pharmaceuticals, and food and beverage processing [3]. Additionally, methyl- and ethyl-PB are added as preservatives in food products [23]. PBs also occur naturally in certain foods, wine, and plants [8].

In vitro studies have shown that PBs induce the growth of MCF-7 human breast cancer cells and influence the expression of estrogen-dependent genes [3,24]. Moreover, there are indications that the estrogenic burden of parabens and their metabolites in blood may exceed the action of endogenous estradiol during childhood [23].

Metabolically, PBs are partially hydrolyzed by esterases to p-hydroxybenzoic acid and subsequently form glycine, glucuronide, or sulfate conjugates. These conjugates have increased water solubility, making them more amenable to urinary excretion than the free species [8].

### 1.3. Phthalate Esters

Phthalate esters (1,2-diesters; PEs) have a wide range of applications. High molecular weight (HMW) phthalates are used as plasticizers in various industrial products, including bags, carpets, building materials, medical equipment, clothing, toys, and food packaging. They are not chemically bound to the plastic and thus are ubiquitous in the environment. Low molecular weight (LMW) phthalates are used in pharmaceuticals, cosmetics, and personal care products such as nail polish and fragrances [16]. Animal studies have associated phthalate exposure with adverse effects on reproductive and developmental health, including an increased risk of cancer and anti-androgenic effects on male reproductive development after prenatal exposure, such as undescended testes, asthma, and decreased testes weight [25,26]. Furthermore, epidemiological studies in humans have linked phthalate exposure to delayed pubarche, reduced anogenital distance, decreased semen quality, and other effects [27,28].

Phthalates typically undergo a two-step metabolic pathway: hydrolysis (phase-I), where the phthalate diester is hydrolyzed into the primary metabolite monoester phthalate, and conjugation (phase-II), forming the more hydrophilic glucuronidated metabolite. In some cases, especially for HMW phthalates, further oxidation occurs before excretion. Conversely, LMW phthalate metabolites, such as mono-ethyl phthalate, may skip the glucuronidation step and be excreted directly in their free form [6]. Many phthalate esters yield phthalic acid as an end-product metabolite, which has been used as a biomarker, although it suffers from very low specificity [29].

### 1.4. Triclosan

Triclosan (TCS) is a biocidal compound with activity against bacteria and fungi, widely used as an additive in various personal care and hygiene products such as cosmetics, deodorants, disinfectants, antiperspirants, soaps, and toothpaste [30]. It has been detected in human blood, urine, breast milk, umbilical cord blood, and amniotic fluid [31,32,33,34]. Furthermore, TCS is widespread in the aquatic environment and has been found in urban effluent waters and surface waters [35,36,37]. TCS is considered more toxic than many other disinfectants and exhibits estrogenic activity [38,39,40]. In experimental animal models, TCS has been shown to act as an endocrine disruptor [41] and to affect thyroid hormone, testosterone, and estrogen levels [42,43,44,45,46]. Moreover, human exposure to TCS has been linked to an increased risk of asthma, cancer, and obesity [47,48]. The human body rapidly metabolizes TCS through hepatic uridine diphosphate-glucuronyltransferase facilitated conjugation to glucuronic acid, after which its glucuronidated form is eliminated in urine [7].

### 1.5. Per- and Polyfluoroalkyl Substances

Per- and polyfluoroalkyl substances (PFAS) comprise a wide range of synthetic fluorinated substances, including oligomers and polymers. To date, estimating the number of PFAS-related compounds remains challenging due to their extensive use in numerous commercial and consumer products. The most recent attempt to estimate the number of compounds in this class was reported in 2018 by the OECD/UNEP Global PFC Group, which identified 4730 CAS numbers as PFAS-related [49]. The difficulty in cataloging these compounds leads to uncertainty regarding the estimated global and regional emissions of such chemicals into the environment. To date, perfluorooctanoic acid (PFOA) and perfluorooctane sulfonate (PFOS) are the two most extensively investigated PFAS.

PFAS are considered potential endocrine-disrupting chemicals (EDCs) [50]. Different PFAS have been associated in vitro with effects on the estrogen receptor (ER), the androgen receptor (AR) [51], and/or the aryl hydrocarbon receptor (AhR) transactivation [52]. As an example, breast cancer epidemiology presents mixed association, with many studies reporting positive associations with PFAS [53], while others present inverse associations [54], as well as no associations [55]. Recently, the Monographs program of the International Agency for Research on Cancer (IARC) evaluated the carcinogenicity of PFOA and PFOS, classifying PFOA as carcinogenic to humans (group 1). The classification was based on the combination of sufficient evidence of cancer in experimental animals and strong mechanistic evidence in exposed humans (epigenetic alteration and immunosuppression). For humans, the evidence for cancer was considered limited, with the most consistent associations observed between PFOA and renal cell carcinoma and testicular cancer. On the other hand, PFOS is classified as possibly carcinogenic to humans (group 2B) with strong mechanistic evidence but limited or inadequate epidemiological and animal data [56,57].

## 2. Comprehensive Bibliographic Search of Endocrine Disruptors Biomonitoring

To broadly and unbiasedly review the existing literature, we conducted a comprehensive bibliographic search for mass spectrometric methods used in determining PBs, PEs, bisphenols, TCS in human urine, and PFAS in blood. Specifically, the literature search for comparable studies was carried in the PubMed database on 2 November 2024. The search and selection were performed by two researchers independently and all authors reviewed the findings.

The search criteria were as follows:

(I) Journal articles with titles containing (“pfas”[Title] OR “perfluoroalkyl*”[Title] OR “phthalate*”[Title] OR “paraben*”[Title] OR “BPA”[Title] OR “bisphenol*”[Title] OR “BPS”[Title] OR “BPP”[Title] OR “BPF”[Title] OR “BPAF”[Title] OR “endocrine disruptor*”[Title]) AND (“urin*”[Title] OR “biomonitoring”[Title] OR “blood”[Title] OR “serum”[Title] OR “plasma”[Title]) AND (“method”[Title] OR “detection”[Title] OR “quantitat*”[Title] OR “determination”[Title] OR “*LC”[Title] OR “GC”[Title] OR “chromatograph*”[Title] OR “mass spectrometr*”[Title] OR “analysis*”[Title] OR “measurement”[Title]).
(II) Publications from 1 January 2004, to October 2025.

The initial search yielded 362 papers. We included research articles written in English that presented mass spectrometric analytical methods for exposure assessment by quantifying total metabolite levels (free, glucuronated, and sulfated) of phthalates, parabens, triclosan, and bisphenols in human urine, or PFAS in blood. Of these, 81 papers met the search criteria and are summarized in Table 1, Table 2 and Table 3.

## 3. Analysis of Endocrine-Disrupting Chemicals in Biological Samples Using Chromatography and Mass Spectrometry Methods Following Human Exposure

Several in vivo studies have been conducted on animals to investigate the health effects of exposure to endocrine disruptors (EDCs). These studies have indicated various potential health effects in humans, but they suffer from a major limitation that makes direct extrapolation to humans difficult. Human exposure is highly complex and challenging to model. EDCs are typically added to mixtures and are present in a wide variety of everyday products, exposing humans to multiple EDCs simultaneously. Since many EDCs share a similar mode of action, such as estrogenic activity, they may induce a biological effect, even at low individual doses [137]. A characteristic example of underestimated exposures is presented by a recent study which revealed that BPA exposure levels exceed the newly updated acceptable daily intake through regular beverage consumption [138]. For the aforementioned reasons, the exposure assessment of multiple classes of endocrine disruptors (EDCs) is very important. To evaluate human exposure to BPA and other bisphenols, PBs, PEs, TCS, and PFAS, it is essential to measure the urinary or blood concentration of free species and their conjugates [7,10,14,18]. For the non-persistent EDCs, human exposure is assessed by the total concentration of metabolites in urine, from which daily intake estimates can be extrapolated by reconstructing the initial exposure based on measured biomarker levels and toxicokinetic parameters [9,10]. For the persistent ones, such as PFAS, the concentrations in blood are indicative of the cumulative exposure [139]. Furthermore, special attention has been given to pregnant women and children, as the endocrine-disrupting effects are more intense during embryonic life and early childhood [1]. Thus, several studies have taken place in human mother–child cohorts where exposure levels are correlated with clinical, nutritional, socio-economic, etc., metadata [9,10,140] to either link with health outcomes or better understand the sources of exposure. Blood and serum analysis is often limited by small sample volumes, especially in pediatric patients, and by significant matrix effects caused by proteins and lipids. For PFAS, validated micro-volume and microsampling techniques (such as dried blood spots and volumetric absorptive microsampling) allow accurate quantification using only tens of microliters [141,142]. These methods have demonstrated good agreement with results obtained from venous serum and whole blood samples.

EDCs are found in very low concentrations in human urine, typically in the low ng/mL range. Moreover, some of them produce multiple metabolites through oxidation processes (e.g., HMW phthalates) [6]. Urine is also a complex matrix with a very high number of metabolites and salts at very high concentrations, a factor that makes the absolute and precise quantification of compounds at trace levels even more challenging [143]. It is a common practice to enzymatically hydrolyze glucuronide and sulfate conjugates in order to measure only the free form of the metabolites and practically estimate the total levels [6]. The analytical challenge here is to effectively remove the enzymes from the samples or use them in minimal amounts because they tend to block the chromatographic columns.

For the reasons outlined above, Liquid Chromatography–Tandem Mass Spectrometry (LC-MS/MS) has been primarily used, with Gas Chromatography–Mass Spectrometry (GC-MS) serving as an alternative method, for the determination of these compounds in human biofluids (see Table 1, Table 2 and Table 3). Through the use of chromatography, the metabolites are separated from any interfering signals, adding an extra dimension of identification beyond mass spectrometric detection, which is often necessary due to the presence of several common isobaric isomers such as, for example, mono-iso and n-butyl phthalates. In LC approaches especially, matrix effects are minimized. Concerning detection, mass spectrometers are employed—these are, more specifically, quadrupoles operated in single-ion-monitoring (SIM) or selected-reaction-monitoring (SRM) modes—due to their superior sensitivity. High-resolution spectrometers have not been extensively used since their sensitivity is insufficient for some metabolites, and the classic untargeted metabolomics approaches that typically follow minimal sample preparation do not allow detailed coverage of most EDCs.

## 4. Liquid Chromatography–Mass Spectrometry-Based Techniques

### 4.1. Sample Preparation

The primary goals of sample preparation in the protocols for human biomonitoring of environmental contaminants [143] are as follows:

(I) Removing solid particles and precipitating proteins to prevent column blockages (mostly in blood matrices). (II) Diluting samples and adjusting pH to enhance chromatographic performance. (III) Spiking with isotopically labeled standards to improve method accuracy. (IV) Removing enzymes used for hydrolyzing conjugates (mainly in urine matrices) to protect chromatographic columns, especially Ultra-Performance Liquid Chromatography (UPLC) columns with smaller particle sizes. (V) Cleaning up salts (either endogenous or added buffers) that suppress ionization and cause intense matrix effects. (VI) Derivatizing poorly ionizable compounds at low levels, such as BPA, to enhance sensitivity [9,59].

**Figure 2:**
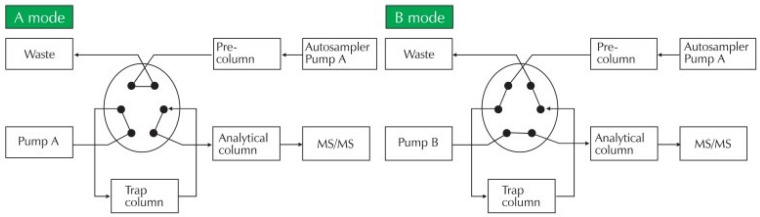
Schemes of switching column procedure. (1) A mode—online cleanup, (2) B mode—concentration to trap-column, (3) A mode—separation by analytical column and injection to MS/MS detector. (Reprinted from Jeong et al., 2011 [61]).

Given the high number of samples in human epidemiological studies, method throughput and simplicity are as important as analytical performance [72]. Automating sample preparation steps, or even the entire protocol, greatly enhances applicability for large-scale studies. For instance, using restricted access materials (RAM) for online cleanup in a column-switching system connected to the LC-MS instrument (Figure 2) [61] or employing within-a-single 96-well plate protein precipitation and sample injection methods [124] can minimize sample preparation steps and reduce consumables and solvent waste.

Blank contamination is typically not a major concern, except in PFAS analyses, where certain solvents, SPE cartridges, or LC-MS tubing and components may introduce PFAS artifacts [124,125]. In contrast to phthalate ester (PE) metabolites, both BPA and TCS are environmentally ubiquitous. Standard bioanalytical laboratory precautions are therefore essential to mitigate contamination risks.

Depending on the endocrine disruptors (EDCs) and study goals, different enzymes can be used for hydrolyzing conjugated species, or samples can be analyzed without hydrolysis to determine only free levels. E. coli β-glucuronidase hydrolyzes glucuronated species, suitable for parabens (PEs), triclosan (TCS), and phthalate metabolites (PBs). However, bisphenol A (BPA) requires H. pomatia β-glucuronidase/sulfatase to hydrolyze both glucuronated and sulfated conjugates [9]. Including enzymatic hydrolysis in every study is advisable to determine total EDC metabolite levels reflecting exposure to parent compounds.

There are three main sample cleanup strategies. One strategy is dilute-and-shoot or protein-precipitation [144]: Samples are diluted with a cold organic solvent (acetonitrile, isopropanol, or methanol) and centrifuged. This approach is fast but may not effectively cleanup salts and can cause matrix effects, requiring more frequent column and ionization source maintenance [143].

The other two strategies are liquid–liquid extraction (LLE) [59] and solid-phase extraction (SPE) [9]. Several miniaturized sample preparation techniques have been proposed as appealing alternatives to address the limitations of conventional extraction methods. These include dispersive liquid–liquid microextraction (DLLME) [76,85], microextraction using packed sorbent (MEPS) [88], ultrasound-assisted emulsification microextraction [94], and vortex-assisted dispersive liquid–liquid microextraction (VADLLME) [97]. These techniques offer enhanced analytical performance by more effectively cleaning up samples, minimizing matrix effects, and reducing instrument maintenance downtime. Nevertheless, solid-phase extraction (SPE) remains the most widely used due to its superior selectivity, efficient sample cleanup, compatibility with liquid chromatography (LC) solvents, and greater potential for automation [127] and online analysis [132].

The most commonly used SPE sorbents are reversed-phase materials, such as C18 and N-vinylpyrrolidone/divinylbenzene copolymers. For PFAS, a large and diverse class of chemicals, different SPE sorbents may yield varying recovery rates for different PFAS species [125]. SPE is generally more expensive, labor-intensive, and requires more method development than liquid–liquid extraction (LLE); however, it has great automation potential and tends to be more reproducible and robust.

Due to intense matrix effects, using isotopically labeled analogs for as many target compounds as possible is a common and strongly recommended practice [143]. This is particularly critical in complex matrices where analyte extraction is challenging, such as dried blood spots (DBS). While isotopic dilution cannot entirely eliminate signal suppression and sensitivity loss, it can effectively correct for these effects, resulting in more robust and accurate measurements.

### 4.2. Instrumental Analysis

LC-MS/MS is the preferred platform for such analyses for several reasons. The most important is that it eliminates the need for derivatization (at least in modern instruments) and water removal from the samples, simplifying the sample preparation process. Most methods employ phenyl or C6-phenyl LC columns, often using HPLC or, in more recent approaches, UPLC. These columns offer excellent separation, with the added advantage of π-π interactions between the aromatic rings of the target analytes and the sorbent material. C18 LC columns have also been used effectively [9,60,72,83]. Generally, both phenyl and C18 columns provide satisfactory separation with conventional HPLC [9], but UPLC facilitates the baseline separation of certain structural isomers (e.g., isopropyl-PB and n-propyl-PB) more easily, often using shorter gradients and therefore ending up in higher throughputs with reduced analytical costs.

**Figure 3:**
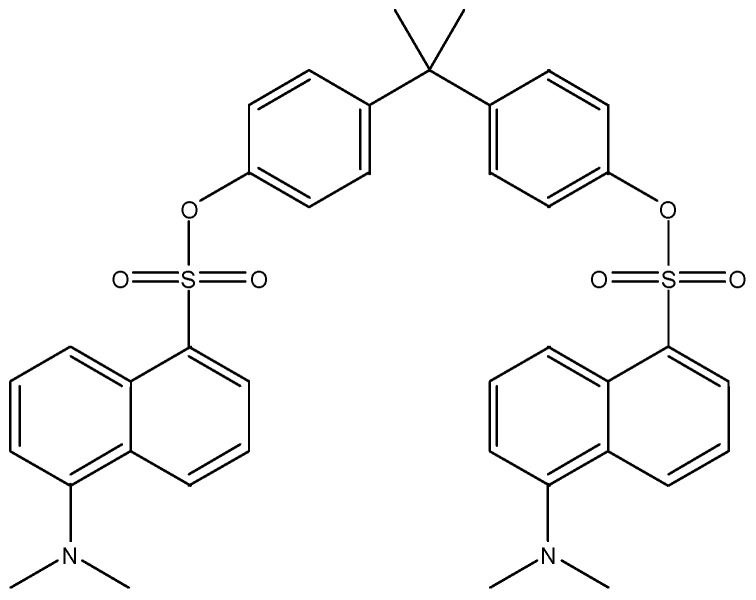
Derivatized BPA with dansyl chloride (reprinted from Myridakis et al., 2015) [9].

**Figure 4:**
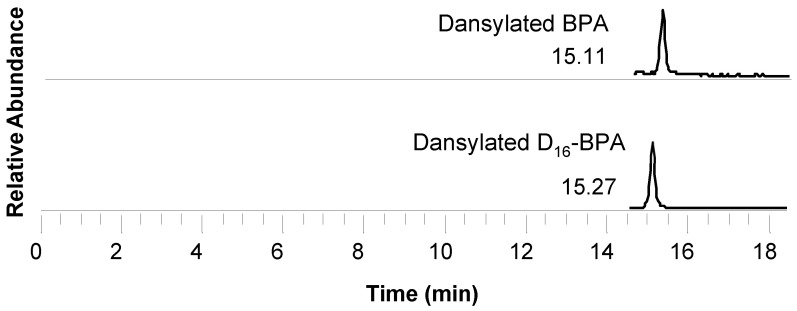
Derivatized BPA—human urine chromatogram (reprinted from Myridakis et al., 2015) [9].

Electrospray ionization in negative mode is the most common ionization technique for these metabolites, as all of them contain labile hydroxyl or carboxyl hydrogens, making them prone to easily forming anions. The mobile phases often include formic or acetic acid, which can partially suppress negative ionization. However, this suppression is necessary to achieve proper retention of the target compounds in the chromatographic column, as many of these compounds are partially ionized at neutral pH. It is important to note that among the studied metabolites, BPA exhibits the poorest ionization and is detected at very low levels, often approaching the detection limits of certain instruments. For this reason, some studies include an additional derivatization step (e.g., forming dansyl analogs, as shown in Figure 3 and Figure 4) which significantly enhances its detection limit (by more than two orders of magnitude). The addition of two highly nucleophilic nitrogen atoms to the BPA molecule substantially improves positive ionization [9,142]. Furthermore, dansyl chloride reacts with any aromatic –OH group, making it applicable in multi-phenol methods (such as for PBs, BPA analogs, and TCS). Although these reactions are straightforward, one-step processes that do not require additional cleanup, they compromise the main advantage of LC over GC, which is the simplicity of sample preparation. The mass spectrometers, almost exclusively triple quadrupoles, operate in SRM mode, offering sufficient selectivity, particularly when combined with chromatography, and adequate sensitivity for the quantitative detection of EDC metabolites. Notably, for certain PFAS species such as PFOS and PFOA, a correlation between absolute plasma concentrations measured using a targeted UPLC-MS/MS method and relative abundances determined through an untargeted metabolomics platform has been reported [136].

The monitored Selected Reaction Monitoring (SRM) transitions for bisphenols, phthalates, parabens, and triclosan, as reported in the cited publications using LC/MS-MS analysis, are summarized in Table 4. For PFAS, the corresponding data are reported in Table 4 as mass-to-charge ratio (*m*/*z*) ranges for both precursor and product ions for each PFAS category, given the large number (twenty-eight) of determined compounds. For a detailed report on the precursor and product ions used for quantitation and confirmation transitions for all analyzed PFAS, refer to Nakayama et al., 2020 [125].

## 5. Gas Chromatography–Mass Spectrometry-Based Techniques

### 5.1. Sample Preparation

The goals for Gas Chromatography–Mass Spectrometry (GC-MS) sample preparation are largely the same as those for LC-MS/MS. The primary difference is the necessity of complete water removal and the protection of labile hydrogens in target molecules to enhance their volatility and thermal stability, making them compatible and detectable with GC [145]. Cleanup steps typically involve liquid–liquid extraction (LLE) [64] or solid-phase extraction (SPE) [110], either in traditional forms or more contemporary variations such as ‘quick, easy, cheap, effective, rugged, and safe’ (QuEChERS) [116], magnetic SPE [107], and dispersive liquid–liquid microextraction (DLLME) [111]. Additionally, derivatization strategies include classic silylation reactions (such as those using N,O-Bis(trimethylsilyl)trifluoroacetamide (BSTFA) [108,109] and other approaches [111,113]. The studied metabolites are readily derivatized, with many well-established derivatization agents (especially the dominating silylating ones) being compatible. The main limitation of these techniques is their challenging automation and online integration with GC-MS systems to enhance method throughput, ensure consistent derivatization immediately prior to injection, and minimize human error. Notably, matrix effects are generally less pronounced in GC compared to electrospray ionization (ESI) in LC [143]; thus, the use of isotopically labeled standards for every target is not always mandatory.

### 5.2. Instrumental Analysis

In most cases, typical low-bleed GC columns are used, such as DB5-MS (phenyl-arylene polymer) [110] and HP5-MS (5%–phenyl-methylpolysiloxane) [111]. Since the samples are quite clean, they are usually injected in the split-less mode [111], where the entire volatile fraction of the injected sample is directed to the column and sensitivity is maximized. However, use of such approaches is more analytically challenging in achieving adequate chromatographic performance compared to the more “forgiving” high split injections. Regarding mass spectrometric analysis, single quadrupoles are used in most cases, operated in Selected Ion Monitoring (SIM) mode and with electron impact (EI) ionization. The target compounds yield characteristic and analyte-specific ions, and in combination with the superior chromatographic resolution of GC, there is no real need for triple quadrupoles or high-resolution mass analyzers. The selected ions monitored for the determination of the studied compounds, as reported in the cited publications using GC/MS-MS analysis, are also summarized in Table 4.

## 6. Conclusions

The bibliographic search reveals that LC-MS is the preferred choice for most analytical laboratories in determining these compounds (all but PFAS where exclusively LC-MS is employed), with 39 published methods (Table 1) compared to 17 GC-MS methods (Table 2). This preference can be attributed to the simplicity of sample preparation, the higher potential for automation and online analysis, and generally higher throughput, a critical parameter in large-scale epidemiological studies involving thousands of samples. Solutions employing Restricted Access Materials (RAM) with online column-switching systems connected to LC-MS instruments are expected to gain further traction [61,72,89] due to their simplicity, throughput, and effectiveness. The use of specialized extraction solvents enables direct, repetitive injection of urine onto these supports. Coupling RAMs with column-switching LC systems offers a highly attractive approach to biological sample preparation [146].

Moreover, Ultra-Performance Liquid Chromatography (UPLC) offers even faster analysis times [83], enhanced separations, and further strengthens the case for LC. Another crucial factor is metabolite coverage. Given the high cost of such analyses and the value of human cohort samples, the ability to analyze multiple categories of endocrine disruptors (EDCs) in a single run is highly desirable. This is especially important as humans are exposed to various EDC mixtures, many of which share similar modes of action [9,72,143]. LC facilitates this multi-class analysis, with numerous methods available (Table 1), compared to only two GC-MS methods (Table 3) [105,110].

This multi-class analysis directly resonates with the broader concept of the human exposome, which seeks to capture the totality of lifetime environmental exposures [147,148]. Embedding biomonitoring methods within this paradigm not only emphasizes their relevance for characterizing complex chemical mixtures but also creates a bridge to downstream approaches, such as multi-omics and pathway analyses, that can reveal the way these exposures translate into biological effects [149].

Despite these advantages, LC is not without limitations. Certain analytes, such as poorly ionizable compounds like BPA, may still pose challenges even with modern triple quadrupole instruments that offer improved sensitivity. In these cases, gas chromatography (GC) remains an attractive alternative, as it is less affected by matrix effects, can achieve selective detection without tandem mass spectrometry, and does not necessitate expensive isotopically labeled standards for each target metabolite.

High-resolution spectrometers like Orbitrap or Time-Of-Flight instruments have not been widely used, except in PFAS analysis, due to insufficient sensitivity for some metabolites and the inability of classic untargeted metabolomics approaches to quantitate these compounds effectively. Nevertheless, with recent advancements in MS technology and continuous improvements in detection limits [150], methods based on untargeted data acquisition with high-resolution or hybrid mass analyzers [151,152] are becoming more appealing. These methods exponentially increase metabolite coverage and enable the concurrent monitoring of known and the discovery of unknown EDC metabolites with applications such as SQUAD, where triple quadrupole-level sensitivity and specificity is combined with untargeted discovery on a high resolution orbitrap analyzer [151], paving the way for the future biomonitoring approaches and exposomics pipelines. Additionally, integrating endogenous metabolomic data acquired from the same samples can provide mechanistic insight into the underlying biology. However, the increased necessity for extensive sample pre-treatment (e.g., conjugate hydrolysis), cleanup (e.g., SPE), and more specific chromatographic conditions (e.g., not 0.1% formic acid in water/acetonitrile for UPLC applications) for the effective trace quantification and elimination of matrix effects, subsequently and substantially alter the metabolome/exposome and remain a challenge for the hybrid approaches.

To the best of our knowledge, no previous review has compared LC-MS and GC-MS biomonitoring methods across all major classes of endocrine disruptors. By consolidating these approaches into a unified framework, this work addresses a critical gap in the literature and provides a practical reference for laboratories seeking to optimize multi-class exposure assessment strategies in large-scale human studies.

## Figures and Tables

**Table 1:** Liquid Chromatography–Mass Spectrometry-based methods for the determination of bisphenols, parabens, phthalate esters, and triclosan.

Reference	Sample Preparation	Ionization	BPA, Other Bisphenols	PBs	PEs	TCS
Markham et al., 2010 [58]	Solid Phase Extraction (SPE)	ESI	BPA	-	-	-
Fox et al., 2011 [59]	Liquid–Liquid Extraction (LLE)	ESI	BPA	-	-	-
Frederiksen et al., 2011 [60]	SPE	ESI	-	5	-	-
Jeong et al., 2011 [61]	Column Switching	ESI	-	-	4	-
Solymos et al., 2011 [62]	Dilute and Shoot	ESI	-	-	3	-
Chen et al., 2012 [63]	SPE	ESI	BPA	-	5	-
Gries et al., 2012 [64]	LLE	ESI	-	-	3	-
Liao & Kannan, 2012 [65]	SPE	ESI	BPA	-	-	-
Monfort et al., 2012 [66]	LLE	ESI	-	-	5	-
Nicolucci et al., 2013 [67]	SPE	ESI	BPA	-	-	-
Wang et al., 2013 [68]	SPE	ESI	BPA	-	14	-
Asimakopoulos et al., 2014 [69]	LLE	ESI	-	7	-	X
Battal et al., 2014 [70]	Protein precipitation	ESI	BPA			
Markham et al., 2014 [71]	SPE	ESI	BPA	-	-	-
Moos et al., 2014 [72]	Protein precipitation—Column Switching	ESI	BPA	9	-	X
Zhou et al., 2014 [73]	SPE	ESI	BPA, 2 others	4	-	X
Li & Franke, 2015 [74]	LLE	ESI	BPA	-	-	-
Myridakis et al., 2015 [9]	SPE	ESI	BPA	6	7	-
Sabaredzovic et al., 2015 [75]	SPE	ESI	-	-	12	-
Rocha et al., 2016 [76]	Dispersive Liquid–Liquid Microextraction (DLLME)	ESI	BPA, 6 others	-	-	-
Wu et al., 2016 [77]	Dual Microextraction	ESI	-	-	3	-
Heffernan et al., 2016 [78]	SPE	ESI	BPA, 4 others	-	14	-
Ren et al., 2016 [79]	SPE	ESI	BPA	5	-	X
Jing et al., 2011 [80]	SPE	ESI	BPA	-	-	-
Servaes et al., 2013 [81]	Dilute and Shoot	ESI	-	-	7	-
Anderson et al., 2014 [82]	LLE	ESI	BPA	-	-	-
Dewalque et al., 2014 [83]	SPE	ESI	-	4	7	-
Provencher et al., 2014 [84]	LLE	ESI	BPA	-	-	X
Vela-Soria et al., 2014 [85]	DLLME	ESI	BPA, 1 other	4	-	-
Venisse et al., 2014 [86]	LLE	ESI	BPA	-	-	-
Buscher et al., 2015 [87]	SPE	ESI	BPA	-	-	-
Cristina Jardim et al., 2015 [88]	Microextraction using Packed Sorbent (MEPS)	ESI	-	5	-	-
Herrero et al., 2015 [89]	Column switching	ESI	-	-	9	-
ter Halle et al., 2015 [90]	Organogel materials	APCI	BPA	-	-	-
Grignon et al., 2016 [91]	SPE	ESI	BPA	-	-	-
Hauck et al., 2016 [92]	SPE	ESI	BPA	-	-	-
Schlittenbauer et al., 2016 [93]	Filtration	ESI	-	8	-	-
Zhou et al., 2018 [94]	Ultrasound-assisted Emulsification Microextraction	ESI	-	9	-	-
Carrasco-Correa et al., 2015 [95]	DLLME	ESI	-	4	-	-
Battal et al., 2021 [96]	LLE	ESI	BPA	-	-	-
Bocato et al., 2020 [97]	Vortex-assisted DLLME (VADLLME)	ESI	BPA, 6 others	7	-	-
Chen et al., 2022 [98]	Supported Liquid Extraction (SLE)	ESI	BPA, 2 others	4	-	X
Frigerio et al., 2020 [99]	SPE	ESI	BPA	-	7	-
Jo et al., 2020 [100]	Online SPE	ESI	BPA, 2 others	-	-	-
Pia Dima et al., 2020 [101]	SPE	ESI	BPA	-	7	-
Sanchis et al., 2019 [102]	Dilute and Shoot	ESI	BPA, 2 others	4	-	-
Silveira et al., 2020 [103]	MEPS	ESI	BPA, 3 others	7		

**Table 2:** Gas Chromatography–Mass Spectrometry-based methods for the determination of bisphenols, parabens, phthalate esters, and triclosan.

Reference	Sample Preparation	Ionization	BPA, Other Bisphenols	PBs	PEs	TCS
Kawaguchi et al., 2008 [104]	Hollow Fiber-Assisted Liquid-phase Microextraction (HF-LPME)	EI	BPA	-	-	-
Geens et al., 2009 [105]	SPE followed by Acidified Silica Purification	ECNI	BPA	-	-	X
Kondo et al., 2010 [106]	Liquid–Liquid Extraction (LLE)	EI	-	-	5	-
Gries et al., 2012 [64]	LLE	EI	-	-	3	-
Rastkari & Ahmadkhaniha, 2013 [107]	Magnetic SPE	EI	-	-	5	-
Kim et al., 2014 [108]	LLE	EI	-	-	8	-
Kubwabo et al., 2014 [109]	Solid Phase Extraction (SPE)	EI	BPA	-	-	-
Azzouz et al., 2016 [110]	SPE	EI	BPA	7	-	X
Pastor-Belda et al., 2016 [111]	Dispersive Liquid–Liquid Microextraction (DLLME)	EI	BPA	-	-	-
Brigante et al., 2017 [112]	SPE	EI	BPA	-	-	-
Hui-Ting et al., 2017 [113]	Optimal Ultrasound-assisted Emulsification Microextraction	EI	-	4	-	-
Yoshida, 2017 [114]	LLE	EI	-	-	9	-
Chung & Ding, 2018 [115]	SPE	EI	BPA	-	-	-
Correia-Sa et al., 2018 [116]	Micro-Quick, Easy, Cheap, Effective, Rugged and Safe (QuEChERS)	EI	BPA	-	-	-
Cunha & Fernandes, 2010 [117]	Dispersive Liquid–Liquid Microextraction (DLLME)	EI	BPA, 1 other	-	-	-
Herrero et al., 2015 [89]	LLE	EI	-	-	9	-
Kawaguchi, Ito, Honda et al., 2008 [104]	Stir Bar Sorptive Extraction (SBSE)	EI	-	-	-	X
Elliani et al., 2020 [118]	Solid Phase Microextraction (SPME)	EI	-	-	7	-
Kucuk et al., 2024 [119]	Dummy molecularly imprinted polymer-based SPE	EI	-	-	3	-
Polovkov et al., 2020 [120]	LLE	EI	BPA	-	-	-

**Table 3:** Liquid Chromatography–Mass Spectrometry-based methods for the determination of PFAS.

Reference	Sample Preparation	Separation	Ionization
Kotlarz et al., 2020 [121]	LLE	UPLC	ESI (Orbi)
Li et al., 2022 [122]	LLE	UPLC	ESI (MS/MS)
Salihovic et al., 2020 [123]	Solid Phase Extraction (SPE)	UPLC	ESI (MS/MS)
Da Silva et al., 2020 [124]	LLE	UPLC	ESI (MS/MS)
Nakayama et al., 2020 [125]	LLE + Solid Phase Extraction (SPE)	UPLC	ESI (MS/MS)
Jurado-Sanchez et al., 2014 [126]	Solid Phase Extraction (SPE)	GC	EI
Huber & Brox, 2015 [127]	Solid Phase Extraction (SPE)	UPLC	ESI (MS/MS)
Ma et al., 2013 [128]	LLE	UPLC	ESI (MS/MS)
Poothong et al., 2017 [129]	LLE + Solid Phase Extraction (SPE)	UPLC	ESI (MS/MS)
Yu et al., 2017 [130]	Solid Phase Extraction (SPE)	HPLC	APCI (MS/MS)
Mosch et al., 2010 [131]	Enzymatic hydrolysis + LLE	UPLC	ESI (MS/MS)
Bartolome et al., 2016 [132]	Solid Phase Extraction (SPE)	UPLC	ESI (MS/MS)
Luque et al., 2012 [133]	SUPRAS-based microextraction	UPLC	ESI (MS/MS)
Salihovic et al., 2013 [134]	Solid Phase Extraction (SPE)	UPLC	ESI (MS/MS)
Jiang et al., 2014 [135]	Solid Phase Extraction (SPE)	HPLC	ESI (MS/MS)
Salihovic et al., 2024 [136]	LLE	UPLC	ESI (MS/MS)

**Table 4:** Selected ions used in LC/MS-MS and GC/MS-MS.

Compounds	Ions for LC/MS-MS		Ions for GC/MS-MS (BSTFA Derivatives)	
	Precursor Ion	Product Ion	Precursor Ion	Product Ion
**Bisphenols**				
BPA	227.2	212.2	372.0	357.0
Dansylated BPA	695.5	171.1	-	-
BPS	248.9	108.0	n.a.	n.a.
BPF	199.0	93.2	n.a.	n.a.
BPZ	267.1	173.0	n.a.	n.a.
BPP	345.1	330.0	n.a.	n.a.
BPAF	335.0	265.9	n.a.	n.a.
BPAP	289.1	273.9	n.a.	n.a.
**Phthalates**				
mEP	193.1	92.1	n.a.	n.a.
mnBP/miBP	221.1	77.1	n.a.	n.a.
mBzP	255.2	105.1	n.a.	n.a.
mEHHP	293.2	121.1	n.a.	n.a.
mEOHP	291.2	121.1	n.a.	n.a.
mEHP	277.2	134.1	n.a.	n.a.
**Parabens**				
MPB	151.1	92.1	224.0	209.0
EPB	165.1	92.1	238.0	223.0
nPPB/isoPPB	179.1	92.1	252.0	237.0
nBPB/isoBPB	193.1	92.1	266.0	251.0
BeP	227.0	92.0	n.a.	n.a.
PeP	207.0	92.0	n.a.	n.a.
HeP	235.0	92.0	n.a.	n.a.
**Antimicrobials**				
Triclosan	287.0	35.0	362.0	347.0
**PFAS**				
PFCAs	213.0–913.0	99.0–868.0	n.a.	n.a.
PFSAs	449.0–526.0	80.0–99.0	n.a.	n.a.
FASAs	512.0–526.0	169.0–219.0	n.a.	n.a.
FTS	327.0–789.0	81.0–307.0	n.a.	n.a.

n.a.: not analyzed.

## Data Availability

Not applicable.

## Abbreviations

APCI	Atmospheric Pressure Chemical Ionization
BPA	Bisphenol-A
BPAF	Bisphenol-AF
BPF	Bisphenol-F
BPP	Bisphenol-P
BPS	Bisphenol-S
BSTFA	N,O-Bis(trimethylsilyl)trifluoroacetamide)
DBS	Dried Blood Spot
DLLME	Dispersive Liquid–Liquid Microextraction
EDCs	Endocrine-Disrupting Chemicals
ECNI	Electron Capture Negative Ionization
EI	Electron Impact Ionization
ESI	Electrospray Ionization
GC	Gas Chromatography
HF-LPME	Hollow Fiber-Assisted Liquid-phase Microextraction
HMW	High Molecular Weight
HPLC	High Performance Liquid Chromatography
LC	Liquid Chromatography
LLE	Liquid–Liquid Extraction
LMW	Low Molecular weight
MD-GC	Multidimensional Gas Chromatography
MEPS	Microextraction using Packed Sorbent
MS	Mass Spectrometry
MS/MS	Tandem Mass Spectrometry
Orbi	Orbitrap
PBs	Parabens
PEs	Phthalate Esters
PFAS	Per-and polyfluoroalkyl substances
PFCAs	Perfluoalkyl carboxylic acids
PFSAs	Perfluoroalkyl sulphonic acids
FASAs	Perfluoroalkane sulphanamids
FTS	Fluorotelomer sulphonic acids
PTV	Programmed Temperature Vaporizing
QuEChERS	Quick, Easy, Cheap, Effective, Rugged and Safe
RAM	Restricted Access Material
SBSS	Stir Bar Sorptive Extraction
SIM	Selected Ion Monitoring
SLE	Supported Liquid Extraction
SPE	Solid Phase Extraction
SPME	Solid Phase MicroExtraction
SRM	Selected Reaction Monitoring
TCS	Triclosan
TD-GC	Thermal Desorption Gas Chromatography
UPLC	Ultra Performance Liquid Chromatography
VADLLME	Vortex-Assisted Dispersive Liquid–Liquid Microextraction
